# Determinants of low birth weight deliveries at five referral hospitals in Western Area Urban district, Sierra Leone

**DOI:** 10.1186/s13052-021-01160-y

**Published:** 2021-10-28

**Authors:** David Kabba Kargbo, Kofi Nyarko, Samuel Sackey, Adolphina Addo-Lartey, Ernest Kenu, Francis Anto

**Affiliations:** 1 Field Epidemiology and Laboratory Training Programme, Accra, Ghana; 2 Department of Epidemiology and Disease Control, School of Public Health, University of Ghana, Accra, Ghana; 3 Field Epidemiology Training Program, Free Town, Sierra Leone

**Keywords:** Low birth weight, Case-control, Referral hospitals, Western area urban, Sierra Leone

## Abstract

**Background:**

Low birth weight (LBW) contributes significantly to infant and child mortality. Each year, about 20 1million deliveries are LBW with 96.5% occurring in developing countries. Whiles the incidence of LBW is reducing in other districts of Sierra Leone, it has been reported to be increasing in the Western Area Urban district. Determining the risk factors in a specific geographic area is important for identifying mothers at risk and thereby for planning and taking appropriate action. The current study sought to identify factors associated with LBW deliveries in the Western Area Urban district of Sierra Leone.

**Methods:**

A hospital-based unmatched 1:2 case-control study was conducted among mothers who delivered live singleton babies from November, 2019 to February, 2020 in five referral health facilities. Mothers were conveniently sampled and sequentially enrolled into the study after delivery. Their antenatal care cards were reviewed and a pre-tested questionnaire administered to the mothers. Data analysis was done using Stata 15.0 and association between maternal socio-demographic, socio-economic, obstetric and lifestyle factors and LBW assessed using bivariable and multivariable logistic regression analyses.

**Results:**

A total of 438 mothers (146 cases and 292 controls), mean age: 24.2 (±5.8) and 26.1 (±5.5) years for cases and controls respectively participated in the study. Multivariable analysis revealed that being unemployed (AoR = 2.52, 95% CI 1.16–5.49, *p* = 0.020), having anaemia during pregnancy (AoR = 3.88, 95% CI 1.90–7.90, *p* <  0.001), having less than 2 years inter-pregnancy interval (AoR = 2.53, 95% CI 1.11–5.73, *p* = 0.026), and smoking cigarettes during pregnancy (AoR = 4.36, 95% CI 1.94–9.80, *p* <  0.001) were significantly associated with having LBW babies.

**Conclusion:**

Factors associated with LBW identified were unemployment, anaemia during pregnancy, < 2 years inter-pregnancy interval and cigarette smoking during pregnancy. Health care providers should screen and sensitize mothers on the risk factors of LBW during antenatal sessions.

**Supplementary Information:**

The online version contains supplementary material available at 10.1186/s13052-021-01160-y.

## Background

Low birth weight (LBW) is of major public health concern especially in low and middle-income countries [1]. It contributes significantly to the burden of childhood diseases and increases mortality and disability in neonates and infants [2]. LBW is the weight of a newborn measuring less than 2.5 kg and is measured immediately after birth [3]. According to Agbor et al. however, it has become increasingly evident that the traditional cut-off value of 2.5 kg advocated by WHO to define LBW may not be generalizable to all settings, as variations have been reported from rural and sub-urban settings of even the same country [4]. In their study in rural Cameroon a cut-off value of 2.7 kg was arrived at which supported an earlier report from a sub-urban setting by Njim et al. [5] that reported a value of 2.6 kg as cut-off for LBW.

Globally, 15 to 20% of total births are LBW representing about 20 million births a year [6]. About 96.5% of LBW deliveries in 2019 occurred in developing countries; accounting for 60–80% of neonatal deaths [7]. The prevalence of LBW varies from region to region and within countries; with Asia accounting for 28%, Africa 13% and Latin America 9% [6].

In Sierra Leone, the pattern of LBW deliveries is unstable. The 2008 and 2013 Sierra Leone Demographic and Health Survey (SLDHS) showed that nationwide, LBW deliveries decreased from 11.0% in 2008 to 7.1% in 2013. However, it rather increased in the Western Area Urban district from 9.5 to 17.5% over the same period [8, 9].

Newborns with LBW are at greater risk of neonatal mortality compared to normal weight babies [7]. Management of LBW deliveries increases health care costs due to long periods of hospitalization. It is estimated that care for extremely LBW babies is about six times more expensive compared to normal weight babies [10]. Evidence also exists that LBW increases the likelihood of developing non-communicable diseases like diabetes and cardiovascular diseases in adulthood [10, 11]. LBW has short term consequences, including respiratory distress, infections and hydrocephalus as well as long-term consequences, including coronary heart disease, diabetes, hypertension, and impaired cognitive function [12].

WHO has made a number of recommendations aimed at reducing the number of LBW infants by 30% between 2012 and 2025. These recommendations include, adequate nutrition for adolescent girls, promotion of smoking cessation during and after pregnancy, community-based packages of care to improve linkage and referral for facility births. Others are, intermittent iron and folic acid supplements for women of reproductive age and adolescent girls, in settings where the prevalence of anaemia is 20% or higher and prevention of malaria during pregnancy [13]. All these are implementable at the community level to reduce low birthweight deliveries and therefore neonatal mortality.

Several factors including early initiation of labour, mother’s lifestyle (e.g. smoking and drinking alcohol), multiple pregnancies, maternal age < 15 years or > 35 years, poverty, infections and chronic illnesses including diabetes and hypertension have been reported to contribute to the delivery of LBW babies [11]. The purpose of the current study was to identify factors contributing to LBW in Sierra Leone to allow for the development of sustainable preventive measures in order to reduce the cost to the health sector. This study, therefore, assessed the socio-demographic, obstetric, maternal, and lifestyle factors associated with LBW babies delivered in five referral hospitals in WAU, Sierra Leone.

## Methods

### Study design

A hospital-based unmatched case-control study was undertaken in five referral hospitals in the Western Area Urban district of Sierra Leone from November, 2019 to February, 2020 among mothers who delivered singleton live babies. For each mother who delivered a LBW baby, two mothers who subsequently delivered normal weight babies in the same hospital were selected as controls.

### Study setting

The study was conducted in five-referral hospitals (Government and Non-governmental organization (NGO) supported) offering free maternal services: Princess Christian Maternity Hospital (PCMH), Lumley Government Hospital, King Harman Road Government Hospital, 34 Military hospital, and Aberdeen Women’s Centre (NGO). All these facilities were located within the capital city, Freetown. In 2019, the WAU district had a projected population of 1.2 million. The district is divided into 20 zones and has five referral hospitals and 71 peripheral health units (PHUs). The district recorded 6112 deliveries in health facilities in 2019.

### Study variables

The independent variables included socio-demographic factors (age of mother, educational level, ethnicity, marital status and religion), socio-economic factors, (family income level and occupation), obstetric factors (maternal BMI, weight, height, parity, gravidity, abortion, ANC visits, gestational age, birth interval and anaemia during pregnancy), maternal health and lifestyle factors (diseases like hypertension, diabetes, heart disease, HIV, syphilis and malaria, alcohol intake, smoking and use of herbal medication). The dependent variable was low birth weight.

### Study participants and eligibility criteria

Mothers who delivered live singleton babies in the five referral hospitals during the study period were eligible to participate in the study. The weight of every newborn was measured within an hour of birth. Mothers with unknown last normal menstrual period, caesarean section birth, congenital deliveries, stillbirths and seriously ill mothers were excluded from the study.

#### Case definition

Mothers who delivered babies weighing less than 2.5 kg and consented to take part in the study were enrolled as cases.

#### Control definition

Mothers with newborns weighing 2.5 kg and above were enrolled as controls.

### Sample size determination

An online OpenEpi software, version three for unmatched case-control studies was used to estimate the required sample size. A minimum detectable odds ratio of two and a percentage control group (birth spacing less than 2 years) exposed (67.6%) from a study conducted in Ethiopia was used [14]. Birth spacing was selected as an independent variable as it gave a higher sample size. A case to control ratio of 1:2 with a 95% confidence level and 80% power was used. The required sample size estimated was 438 (146 cases and 292 controls).

### Sampling procedure

A total of 251 singleton live LBW babies were delivered in the five hospitals from November, 2018 to February, 2019 as follows: PCMH:138; King Harman Road Government Hospital: 3; Lumley Government Hospital: 15; 34 Military hospital: 8; and Aberdeen Women’s Centre: 87. The sample size was distributed proportionately among the five facilities based on the November, 2018 to February, 2019 singleton LBW deliveries per facility. The convenience sampling method was used, during which mothers were enrolled sequentially as they delivered until the required sample size for the facility was attained. For each mother who delivered a LBW baby, two mothers who subsequently delivered normal weight babies in the same hospital were selected as controls. In a situation where a case or control declined consent to be part of the study, the next eligible mother was selected as a replacement.

### Data collection tools and procedure

A questionnaire was developed specifically for this study based on the objectives of the study and used for data collection from mothers who met the inclusion criteria of the study. The questionnaire was in three sections: socio-demographic and socio-economic, obstetric, and maternal health and lifestyle factors. The ANC cards of the mothers were reviewed and data on obstetric and maternal health factors extracted. Fifteen trained research assistants (three per hospital and one per shift) collected the data through face-to-face interviews using the common language used in the study area, ‘Creole’. Midwives took the weights of newborn babies within one hour after delivery using calibrated Seca 354 weighing scales (seca gmbh & co.kg 22,089 Hamburg, Germany). These weights, were extracted from the ANC cards of the mothers. The research assistants administered the questionnaires to mothers within 24 h post-delivery in a separate office within the ward to maintain privacy. Data were collected simultaneously in the five hospitals until the required sample size was obtained.

### Quality control

The questionnaire was developed specifically for this study based on the objectives of the study, known variables from literature and the research experience of the authors. The questionnaire was pre-tested in three Government and NGO supported hospitals in the WAU district with similar service characteristics using 2% (three cases and six controls) of the sample size (cases). Findings of the pilot study were used to finalize the questionnaire. The data collected were checked daily for completeness and accuracy by a supervisor. The data were cleaned and entered in SPSS version 22 software and password protected.

### Data processing and analysis

Statistical analysis was done using Stata 15.0 (Stata Corp, College Station, TX USA). A histogram-normal curve was used to check the normality of continuous variables. Descriptive analysis i.e. summary statistics, mean (SD) and proportions were estimated, and inferential analysis using chi-square and logistic regression (bivariable and multivariable) to determine association between LBW and the independent variables. A stepwise backward elimination method with a restricted alpha level of 0.1(10%) was conducted and the post estimation command (“testparm i.variablename”) used to determine variables that met the criteria for the multivariable logistic model. Odds ratio (OR) with 95% CI was computed and statistical significance was determined at *p*-value < 0.05.

### Ethical consideration

Ethical clearance was obtained from the Sierra Leone Ethics and Scientific Review Committee (SLESRC). Permission was also obtained from the Chief Medical Officer, Ministry of Health and Sanitation (MoHS), and the hospital administration. The possible risk of the study (time) was explained to the mothers and their worries addressed. Written informed consent was obtained from each mother or her parent or guardian (mothers less than 18 years old) before data collection. The information collected was treated as confidential and codes were used to identify participants and not names.

## Results

### Socio-demographic characteristics of the mothers

A total of 438 mothers (146 cases and 292 controls) were enrolled into the study from the five-referral hospitals. They were aged 14–39 years, with mean age of 24.2 (SD: 5.8) years for cases and 26.1 (SD: 5.5) years for controls (*p* = 0.685). One hundred and sixty-two of the mothers (36.9%) had no formal education, 25.8% (113) were unemployed and 29.7% (130) were not married (Table 1).

**Table 1:** Socio-demographic characteristics of the mothers, WAU, 2020

Characteristics	Cases (%)	Controls (%)	Total (%)
	***n*** = 146	***n*** = 292	438
**Mother’s age (years)**			
< 20	36 (24.6)	30 (10.3)	66 (15.1)
20–29	82 (56.2)	190 (65.1)	272 (62.1)
≥ 30	28 (19.2)	72 (24.6)	100 (22.8)
**Education**			
No formal education	64 (43.8)	98 (33.6)	162 (36.9)
Primary	23 (15.8)	40 (13.7)	63 (14.4)
Secondary	42 (28.8)	113 (38.7)	155 (35.4)
Tertiary	17 (11.6)	41 (14.0)	58 (13.2)
**Employment status**			
Unemployed	63 (43.1)	50 (17.1)	113 (25.8)
Student	20 (13.7)	39 (13.4)	59 (13.5)
Employed	63 (43.2)	203 (69.5)	266 (60.7)
**Mother/household monthly income**			
< Le 500,000	121 (82.9)	226 (77.4)	347 (79.2)
≥ Le 500,000	25 (17.1)	66 (22.6)	91 (20.8)
**Marital status**			
Single	55 (37.7)	75 (25.7)	130 (29.7)
Cohabiting/married husband unemployed	15 (10.3)	15 (5.1)	30 (6.8)
Cohabiting/married husband employed	76 (52.0)	202 (69.2)	278 (63.5)
**Religious affiliation**			
Christian	60 (41.1)	110 (37.7)	170 (38.8)
Muslim	86 (58.9)	182 (62.3)	268 (61.2)

Bivariable logistic regression analysis of the socio-demographic characteristics of the mothers revealed statistical differences between cases and controls in terms of age (CoR = 3.08, 95% CI 1.60–5.92, *p* = 0.001), employment (CoR = 4.06, 95% CI 2.54–6.47, *p* <  0.001), and marital status (CoR = 1.94, 95% CI 1.25–3.01, *p* = 0.003) (Table 2).

**Table 2:** Comparison of socio-demographic and socio-economic characteristics of mothers of cases and controls, WAU, 2020

Characteristics	Cases (***n*** = 146)	Controls (***n*** = 292)	Crude OR (95% CI)	***p***-value
**Mother’s age (years)**				
< 20	36	30	3.08 (1.60–5.92)	0.001*
20–29	82	190	1.10 (0.66–1.84)	0.688
≥ 30	28	72	1	
**Mother’s highest education**				
No formal education	64	98	1.57 (0.82–3.00)	0.169
Primary	23	40	1.38 (0.64–2.97)	0.401
Secondary/Tec-Voc	42	113	0.89 (0.45–1.74)	0.748
Tertiary	17	41	1	
**Mother’s employment**				
Unemployed	63	50	4.06 (2.54–6.47)	< 0.001*
Student	20	39	1.65 (0.89–3.03)	0.106
Employed/self-employed	63	203	1	
**Mother/household monthly income**				
< Le 500,000	121	226	1.41 (0.83–2.46)	0.182
≥ Le 500,000	25	66	1	
**Marital status**				
Single	55	75	1.94 (1.25–3.01)	0.003*
Cohabiting/married husband unemployed	15	15	2.65 (1.23–5.69)	0.012*
Cohabiting/married husband employed	76	202	1	
**Religious affiliation**				
Christian	60	110	1.15 (0.75–1.76)	0.488
Muslim	86	182	1	

1 = Reference group; *p-value < 0.05 is statistically significant; Crude odd ratio (OR), CI (confidence interval)

### Obstetric characteristics of the mothers

Concerning the obstetric profile, shorter mothers were observed to be higher in cases than controls (32.2% vs 7.8%, p = 0.001). A higher proportion of case mothers made less than four ANC visits compared to control mothers (41.1% vs 20.5%, *p* <  0.001). The mothers who had anaemia during pregnancy were significantly higher among cases than controls (56.2% vs 21.9%, *p* <  0.001). Mothers who had less than 2 years inter-pregnancy interval were observed to be higher among cases than controls (29.3% vs 15.4%,*p* = 0.008). There was no significant difference in the proportion of mothers who had a history of abortion between cases and controls (28.8% vs 25.0, *p* = 0.398). Table 3 displays the obstetric factors found to be associated with delivery of low birth weight babies after bivariable analysis. These factors included, maternal height (CoR = 2.19, 95% CI 1.34–3.55, *p* = 0.001), gravidity (CoR = 1.81, 95% CI 1.18–2.76, *p* = 0.003), number of ANC visits made before delivery (CoR = 2.69, 95% CI 1.70–4.26, *p* <  0.001), and gestational age at delivery (CoR = 12.58, 95% CI 1.66–94.84, *p* = 0.014). Other factors are anaemia during pregnancy (CoR = 4.56, 95% CI 2.90–7.16, *p* <  0.001), and birth spacing (CoR = 2.26, 95% CI 1.15–4.39, *p* = 0.008).

**Table 3:** Obstetric factors associated with delivery of low birth weight babies, WAU, 2020

Characteristics	Cases (***n*** = 146)	Controls (***n*** = 292)	Crude OR (95% CI)	***p***-value
**Mother’s weight (kg)**				
< 50	4 (2.7)	9 (3.1)	0.88 (0.19–3.24)	0.842
≥ 50	142 (97.3)	283 (96.9)	1	
**Mother’s height (m)**				
< 1.5 (short)	47 (32.2)	52 (17.8)	2.19 (1.34–3.55)	0.001*
≥ 1.5 (normal)	99 (67.8)	240 (82.2)	1	
**Mother’s BMI (kg/m**^**2**^**)**				
< 18.5 (underweight)	4 (2.7)	9 (3.1)	1.26 (0.35–4.45)	0.715
18.5–24.9 (normal)	35 (23.9)	48 (16.4)	2.07 (1.11–3.87	0.022
25–29.9 (overweight)	81 (55.5)	161 (55.1)	1.43 (0.85–2.40)	0.176
≥ 30 (obese)	26 (17.8)	74 (25.3)	1	
**Parity**				
Primiparous	83 (56.8)	148 (50.7)	1.28 (0.84–1.95)	0.223
Multiparous	63 (43.2)	144 (49.3)	1	
**Gravidity**				
Primigravida	80 (54.8)	117 (40.1)	1.81 (1.18–2.76)	0.003*
Multigravida	66 (45.2)	175 (59.9)	1	
**ANC Visits**				
< 4 times	60 (41.1)	60 (20.5)	2.69 (1.70–4.26)	< 0.001*
≥ 4 times	86 (58.9)	232 (79.5)	1	
**Gestational age (weeks)**				
Preterm (< 37)	134 (91.8)	213 (72.9)	12.58 (1.66–94.84)	0.014*
Term (37–40)	11 (7.5)	59 (20.2)	3.72 (0.45–30.72)	0.221
Post term (> 40)	1 (0.7)	20 (6.9)	1	
**Anaemia during pregnancy**				
Anaemic (Hb < 11.0 g/dl)	82 (56.2)	64 (21.9)	4.56 (2.90–7.16)	< 0.001*
Not anaemic (Hb ≥ 11.0 g/dl)	64 (43.8)	228 (78.1)	1	
**Birth spacing**				
< 2 years	24 (29.3)	29 (15.4)	2.26 (1.15–4.39)	0.008*
≥ 2 years	58 (70.7)	159 (84.6)	1	
**Previous abortion**				
Ever had	42 (28.8)	73 (25.0)	1.21 (0.75–1.93)	0.398
Never had	104 (71.2)	219 (75.0)	1	

1 = Reference group; *p-value < 0.05 is statistically significant

### Maternal health and lifestyle factors

Hypertension during pregnancy was higher in case mothers than control mothers (32.2% vs 9.6%, *p* <  0.001). Mothers with malaria infection during pregnancy were more seen in cases compared to the controls (72.6% vs 46.9%, *p* <  0.001). Mothers who smoked cigarette during pregnancy were higher in cases compared to the control (35.6% vs 9.9%, *p* <  0.001). A higher proportion of mothers who took herbal medicine during pregnancy were significantly higher in cases than controls (54.8% vs 26.7%, *p* <  0.001). Bivariable analysis showed statistically significant differences between cases and controls in terms of maternal health factors: hypertension status (CoR = 4.47, 95% CI 2.57–7.83, *p* <  0.001), HIV status (CoR = 3.31, 95% CI 1.14–10.29, *p* = 0.015), syphilis infection (CoR = 3.87, 95% CI 2.01–7.55, *p* <  0.001), malaria during pregnancy (CoR = 2.99, 95% CI 1.91–4.73, *p* <  0.001), duration of iron and folic acid use (CoR = 3.55, 95% CI 1.92–6.60, *p* <  0.001). The lifestyle factors associated with delivery of LBW babies were: smoking or living with a partner who smokes (CoR = 5.01, 95% CI 2.92–8.67, *p* <  0.001) and (CoR = 2.31, 95% CI 1.49–3.56, *p* <  0.001) respectively, and taking herbal medicines during pregnancy (CoR = 3.32, 95% CI 2.14–5.15, *p* <  0.001) (Table 4).

**Table 4:** Maternal health and lifestyle factors associated with delivery of LBW babies, WAU, 2020

Determinants	Cases n(%)	Controls n(%)	Crude OR (95% CI)	***P***-value
**Diabetes**				
Diabetic	3 (2.1)	18 (6.2)	0.31 (0.05–1.12)	0.057
Not Diabetic	143 (97.9)	274 (93.8)	1	
**Hypertension**				
Hypertensive	47 (32.2)	28 (9.6)	4.47 (2.57–7.83)	< 0.001*
Not hypertensive	99 (67.8)	264 (90.4)	1	
**Heart disease**				
Heart disease	3 (2.1)	5 (1.7)	1.20 (0.18–6.28)	0.800
No Heart disease	143 (97.9)	287 (98.3)	1	
**HIV**				
Positive	11 (7.5)	7 (2.4)	3.31 (1.14–10.29)	0.010*
Negative	135 (92.5)	285 (97.6)	1	
**Syphilis**				
Positive	31 (21.2)	19 (6.5)	3.87 (2.01–7.55)	< 0.001*
Negative	115 (78.8)	273 (93.5)	1	
**Malaria**				
Positive	106 (72.6)	137 (46.9)	2.99 (1.91–4.73)	< 0.001*
Negative	40 (27.4)	155 (53.1)	1	
**Iron & folic acid used**				
< 3 months	34 (23.3)	23 (7.9)	3.55 (1.92–6.60)	< 0.001*
≥ 3 months	112 (76.7)	269 (92.1)	1	
**Alcohol use**				
Takes alcohol	17 (11.6)	23 (7.9)	1.54 (0.74–3.13)	0.197
Does not take	129 (88.4)	269 (92.1)	1	
**Smoking**				
Smokes	52 (35.6)	29 (9.9)	5.01 (2.92–8.67)	< 0.001*
Do not smoke	94 (64.4)	263 (90.1)	1	
**Living with partner that smoked**				
Partner smokes	68 (46.6)	80 (27.4)	2.31 (1.49–3.56)	< 0.001*
Partner does not smoke	78 (53.4)	212 (72.6)	1	
**Herbal intake during pregnancy**				
Takes	80 (54.8)	78 (26.7)	3.32 (2.14–5.15)	< 0.001*
Not take	66 (45.2)	214 (73.3)	1	

* p-value < 0.05 is statistically significant; AoR (Adjusted odd Ratio)

### Determinates of delivery of LBW babies

After multivariable logistic regression analysis and controlling for possible confounders, factors found to be significantly associated with delivery of LBW babies were: unemployment (AoR = 2.52, 95% CI 1.16–5.49, *p* = 0.020) (Table 5), anaemia during pregnancy (AoR = 3.88, 95% CI 1.90–7.90, *p* <  0.001), having less than two years inter-pregnancy interval (AoR = 2.53, 95% CI 1.11–5.73, *p* = 0.026) and cigarette smoking during pregnancy (AoR = 4.36, 95% CI 1.94–9.80, *p* <  0.001) (Table 6). Even though bivariable analysis showed statistically significant differences between cases and controls in terms of maternal age, marital status, height, gravidity and gestational age at delivery, these factors were no more significantly associated with delivery of LBW babies after multivariable logistic regression analysis (Table 5). Similarly, factors such as hypertension status, HIV status, syphilis infection, malaria during pregnancy, duration of iron and folic acid use, were no more significantly associated with delivery of LBW babies after multivariable logistic regression analysis (Table 6).

**Table 5:** Determinants of delivery of LBW babies, WAU, 2020

Determinants	Cases (*n* = 146)	Controls (*n* = 292)	CoR	*p*-value	AoR	*p*-value
			(95% CI)		(95% CI)	
**Mother’s age (years)**						
< 20	36	30	3.08 (1.60–5.92)	0.001*	1.75 (0.33–9.25)	0.509
20–29	82	190	1.10 (0.66–1.84)	0.688	1.11 (0.52–2.37)	0.778
≥ 30	28	72	1		1	
**Mother employment**						
Unemployed	63	50	4.06 (2.54–6.47)	< 0.001*	2.52 (1.16–5.49)	0.020*
Student	20	39	1.65 (0.89–3.03)	0.106	2.67 (0.93–7.64)	0.066
Employed/self-employed	63	203	1		1	
**Marital status**						
Single	55	75	1.94 (1.25–3.01)	0.003*	1.35 (0.58–3.16)	0.487
Cohabiting/married husband unemployed	15	15	2.65 (1.23–5.69)	0.012*	2.01 (0.57–7.06)	0.276
Cohabiting/married husband employed	76	202	1		1	
**Mother’s height (m)**						
< 1.5 (short)	47	52	2.19 (1.34–3.55)	0.001*	1.52 (0.68–3.37)	0.303
≥ 1.5 (normal)	99	240	1		1	
**Gravidity**						
Primigravida(1)	80	117	1.81 (1.18–2.76)	0.003*	1.77 (0.80–3.90)	0.152
Multigravida(> 1)	66	175	1		1	
**ANC Visits**						
< 4 times	60	60	2.69 (1.70–4.26)	< 0.001*	1.07 (0.49–2.30)	0.870
≥ 4 times	86	232	1		1	
**Gestational age (weeks)**						
Preterm (< 37)	134	213	12.58 (1.66–94.84)	0.014*	4.07 (0.48–34.03)	0.195
Term (37–40)	11	59	3.72 (0.45–30.72)	0.221	2.60 (0.27–25.45)	0.410
Post term (> 40)	1	20	1		1	

1 = Reference group; *p-value < 0.05 is statistically significant

**Table 6:** Determinants of delivery of LBW babies, WAU, 2020

Determinants	Cases	Controls	CoR	*p*-value	AoR	*p*-value
	(*n* = 146)	(*n* = 292)	(95% CI)		(95% CI)	
**Mother anaemia status in pregnancy**						
Anaemic (Hb < 11.0 g/dl)	82	64	4.56 (2.90–7.16)	< 0.001*	3.88 (1.90–7.90)	< 0.001*
Not anaemic (Hb ≥ 11.0 g/dl)	64	228	1		1	
**Birth spacing**						
< 2 years	24	29	2.26 (1.15–4.39)	0.008*	2.53 (1.11–5.73)	0.026*
≥ 2 years	58	159	1		1	
**Hypertension**						
Hypertensive	47	28	4.47 (2.57–7.83)	< 0.001*	1.45 (0.63–3.36)	0.387
Not hypertensive	99	264	1		1	
**HIV**						
Positive	11	7	3.31 (1.14–10.29)	0.010*	4.01 (0.92–17.32)	0.063
Negative	135	285	1		1	
**Syphilis**						
Positive	31	19	3.87 (2.01–7.55)	< 0.001*	1.65 (0.55–4.92)	0.368
Negative	115	273	1		1	
**Malaria**						
Positive	106	137	2.99 (1.91–4.73)	< 0.001*	1.80 (0.88–3.65)	0.103
Negative	40	155	1		1	
**Iron & folic acid used**						
< 3 months	34	23	3.55 (1.92–6.60)	< 0.001*	1.04 (0.39–2.77)	0.933
≥ 3 months	112	269	1		1	
**Smoking**						
Smokes	52	29	5.01 (2.92–8.67)	< 0.001*	4.36 (1.94–9.80)	< 0.001*
Does not smoke	94	263	1		1	
**Living with partner that smoked**						
Partner smokes	68	80	2.31 (1.49–3.56)	< 0.001*	0.91 (0.45–1.84)	0.798
Partner does not smoke	78	212	1		1	
**Take herbal medicine**						
Takes	80	78	3.32 (2.14–5.15)	< 0.001*	1.92 (0.99–3.72)	0.051
Does not take	66	214	1		1	

1 = Reference group; *p-value < 0.05 is statistically significant

## Discussion

An unmatched case-control study was conducted among women who delivered in five referral hospitals in the Western Area Urban district of Sierra Leone to determine factors associated with delivery of low birth weight (LBW) babies. This study has identified some socio-demographic, socio-economic, obstetric and lifestyle-related factors that are associated with the delivery of LBW babies in the study area.

Earlier studies from across Africa have reported some association between the delivery of LBW babies and several maternal socio-demographic, socio-economic, obstetric and medical factors [15–17]. In our current study, mothers who were unemployed were found to have a two-fold odds of giving birth to a LBW baby. This is consistent with findings of a population-based study in southern rural Ghana, which established a strong association between birth weight and maternal employment status. Mothers who were unemployed and coming from the poorest households were reported to be more likely to give birth to LBW babies compared to those who were gainfully employed [15]. Unemployment can contribute to poverty, leading to poor maternal nutritional intake [17]. According to Ahmed, et al., maternal undernutrition and inadequate dietary diversity during pregnancy are significant determinants of delivery of low birth weight babies [18].

The odds of delivering a LBW baby as observed in the current study, was higher among mothers who were anaemic during pregnancy (Hb < 11.0 g/dl), compared to those who were not, similar to an earlier study also carried out in Ghana [12]. Other studies done in Ethiopia [7], Democratic Republic of Congo [17] and India [19] have also reported similar findings in which anaemia during pregnancy was found to be associated with LBW delivery. Anaemia during pregnancy can limit maternal oxygen uptake, thus reducing oxygen supply to the foetus and thus contribute to foetal growth restriction [20].

In this study, babies born within less than 2 years after another child had higher odds of being of LBW, compared with those born after two or more years. Similar findings have been reported from Ethiopia [21, 22] and India [23]. This might be as a result of insufficient replacement of maternal nutrients used-up during the previous pregnancy and this may lead to reduced foetal development. A similar observation made in South Ethiopia by Mingude, et al. [16] has been explained on the basis that, the short inter-pregnancy interval is not able to allow the mother enough time to recover from the nutritional burden and stress of the previous pregnancy, resulting in maternal nutrition depletion. The short inter-pregnancy interval is also associated with maternal iron and folic acid depletion [18]. This reduces the ability of the mother to support foetal growth and development which in turn increases the possibility of growth restriction and LBW in subsequent pregnancies.

Our study also revealed that mothers who smoke cigarettes have higher odds of giving birth to a LBW baby. In a similar case-control study conducted in China [24], pregnant women who were exposed to even passive smoking had an increased risk of delivering low birth weight babies. Although, our study failed to determine the number of cigarette sticks smoked per day and for how long, other studies have reported that, mothers who are heavy smokers (> 8–10 cigarettes/day) had a higher odds of LBW babies [25]. Cigarette smoking is known to reduce oxygen supply to the foetus in-utero as carbon monoxide and nicotine-associated vasoconstriction reduces uterine and placental blood flow, thereby restricting the growth of the foetus, and hence can contribute to LBW [26]. Other factors including gravidity, maternal ill-health and taking of herbal medicine during pregnancy were not found to be significantly associated with the delivery of LBW babies in the current study after multivariable logistic regression analysis.

Even as our current study has identified four main maternal factors (unemployment, anaemia during pregnancy, short inter-pregnancy interval and smoking) as risk factors for the delivery of LBW babies in our study setting, Agbor et al. [4] found only one risk factor for LBW. In their review of eight years records of two health facilities in rural Cameroon, only preterm delivery was found to be associated LBW. Since a higher cut-off value (2.7 kg) was used to define LBW in their study, it was expected that more babies will be caught in the bracket of LBW (an additional 6.1%) and possibly more risk factors identified. This difference in risk factors identified might be due to differences in the study settings. In an earlier study by Njim and colleagues also in Cameroon but in a sub-urban setting, many more risk factors were identified in addition to preterm delivery. Many factors are known to influence the duration of gestation and foetal growth, and thus, the birthweight [27]. These factors may relate to the mother or the physical environment and play an important role in determining the birthweight and the future health of the infant.

According to Johnson and colleagues [28] the risk factors for LBW are interrelated and that exposure to one factor increases the likelihood of exposure to a group of other factors which further increase the risk. Therefore, in addressing the issue of LBW, the risk factors should be tackled together using multi-component interventions. Our current study has identified two important modifiable risk factors for LBW (< 2 years inter-pregnancy interval and cigarette smoking during pregnancy) in addition to anaemia during pregnancy.

These three factors, can be adequately addressed through the implementation of recommendations already made by the WHO [13]. In order to achieve the goal of 30% reduction in the number of LBW infants between 2012 and 2025, WHO has recommended adequate nutrition for adolescent girls and the promotion of smoking cessation during and after pregnancy. Other recommendations include, intermittent iron and folic acid supplements for women of reproductive age and adolescent girls, prevention of malaria during pregnancy and adequate birth spacing.

In addition to iron and folic acid supplements, the issue of anaemia can be addressed through adequate nutrition using locally available foods, including fruits and vegetables. Education during antenatal sessions by attending midwives and dieticians can help in this direction. Prevention of malaria during pregnancy through the use insecticidal nets and intermittent preventive treatment with sulfadoxine-pyrimethamine (IPTp-SP) are also WHO recommendations to improve pregnancy outcomes in moderate to high malaria transmission areas in Africa.

Other interventions that will bring health care closer to the door-steps of pregnant women such as prenatal home-visitation that will focus on social support, health education and access to services, modelled similar to the community-based health planning and services (CHPS) concept in Ghana [29] can contribute to reducing LBW deliveries among at-risk women and adolescent girls in Sierra Leone. Using the CHPS concept, family planning can be promoted to widen the inter-pregnancy interval. Empowering women through job opportunities and formal education can also help reduce LBW, however these may take time to provide considering the current economic situation the world over and the peculiar Sierra Leonean situation where there is the need to recover economically from the 11 years civil war.

Although we employed a case-control study, which we consider an appropriate design for the study, the study is not without limitations as we did not match the cases and controls, which may introduce some selection bias. Assessment of some of the independent variables was liable to recall bias. The measurement we used for maternal smoking may not be the most suitable. Measuring the number of cigarettes sticks smoked by a mother in a day may better show the difference between the cases and controls. Also, the cut-off of 2.5 kg for LBW used in our assessment could exclude some babies from receiving critical clinical care available for LBW babies. These limitations notwithstanding, the study has identified important maternal factors that when addressed, can reduce the incidence of LBW deliveries in Sierra Leone and other developing countries with similar characteristics. The inclusion of all the referral facilities in the city strengthens our design as participants can better represent women of reproductive age in the catchment area of the health facilities.

## Conclusions

This study has established that unemployment, anaemia during pregnancy, less than 2 years of inter-pregnancy interval, and cigarette smoking during pregnancy are significant factors for the delivery of LBW babies. There is a need for health care managers in WAU district to intensify screening and sensitization on the risk factors of LBW during antenatal sessions to address the identified factors among mothers. Also, the Ministry of Youth Affairs should design welfare programmes that would reduce the high rate of unemployment among women in Sierra Leone.

## Supplementary Information

**Additional file 1.** Determinants of LBW in Western Area Urban District, Sierra Leone. Data on which findings of the study was based.

## Data Availability

All data generated during the current study are included in this published article and its additional file.

## Abbreviations

ANC: Antenatal care
AOR: Adjusted Odd Ratio
BMI: Body mass index
CI: Confidence Interval
COR: Crude Odd Ratio
GFELTP: Ghana Field Epidemiology and Laboratory Training Programme
HIV: Human immunodeficiency virus
LBW: Low birth weight
NGO: Non-Governmental organization
PCMH: Princess Christian Maternity Hospital
SD: Standard deviation
SLDHS: Sierra Leone demographic health survey
SLESRC: Sierra Leone Ethics and Scientific Review Committee
SLFETP: Sierra Leone Field Epidemiology Training Programme
SPSS: Software package for social sciences
WAHO: West African Health Organization
WAU: Western Area Urban
WHO: World Health Organization
